# Nutrient salvaging and metabolism by the intracellular pathogen *Legionella pneumophila*

**DOI:** 10.3389/fcimb.2014.00012

**Published:** 2014-02-11

**Authors:** Maris V. Fonseca, Michele S. Swanson

**Affiliations:** ^1^Science and Mathematics Division, Monroe County Community CollegeMonroe, MI, USA; ^2^Department of Microbiology and Immunology, University of Michigan Medical SchoolAnn Arbor, MI, USA

**Keywords:** *Legionella pneumophila*, differentiation, intracellular metabolism, metabolic cues, phagosomal transporters

## Abstract

The Gram-negative bacterium *Legionella pneumophila* is ubiquitous in freshwater environments as a free-swimming organism, resident of biofilms, or parasite of protozoa. If the bacterium is aerosolized and inhaled by a susceptible human host, it can infect alveolar macrophages and cause a severe pneumonia known as Legionnaires' disease. A sophisticated cell differentiation program equips *L. pneumophila* to persist in both extracellular and intracellular niches. During its life cycle, *L. pneumophila* alternates between at least two distinct forms: a transmissive form equipped to infect host cells and evade lysosomal degradation, and a replicative form that multiplies within a phagosomal compartment that it has retooled to its advantage. The efficient changeover between transmissive and replicative states is fundamental to *L. pneumophila's* fitness as an intracellular pathogen. The transmission and replication programs of *L. pneumophila* are governed by a number of metabolic cues that signal whether conditions are favorable for replication or instead trigger escape from a spent host. Several lines of experimental evidence gathered over the past decade establish strong links between metabolism, cellular differentiation, and virulence of *L. pneumophila*. Herein, we focus on current knowledge of the metabolic components employed by intracellular *L. pneumophila* for cell differentiation, nutrient salvaging and utilization of host factors. Specifically, we highlight the metabolic cues that are coupled to bacterial differentiation, nutrient acquisition systems, and the strategies utilized by *L. pneumophila* to exploit host metabolites for intracellular replication.

## Introduction

**Figure 1:**
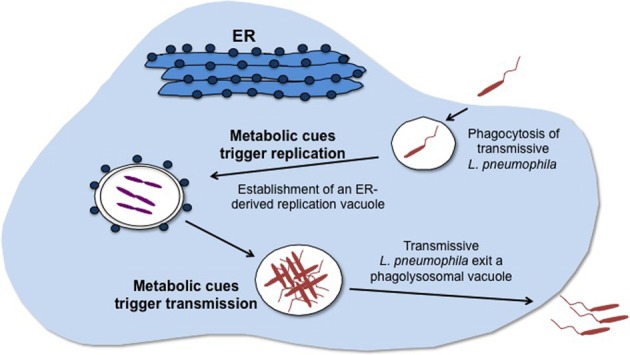
**Metabolic cues govern *L. pneumophila* cellular differentiation.** The infectious, motile, transmissive form of *L. pneumophila* enters its host by phagocytosis. Nutrient abundance is signaled by metabolites, such as amino acids, which trigger differentiation of *L. pneumophila* to the replicative form that then multiplies in an ER-derived vacuolar compartment. Nutritional starvation, signaled by accumulation of particular metabolites, activates the stringent response and a regulatory cascade that coordinates differentiation of *L. pneumophila* to the transmissive form, which seeks a new site favorable for replication.

The facultative intracellular bacterium *Legionella pneumophila* parasitizes protozoa in aquatic environments and alveolar macrophages in susceptible human hosts. *L. pneumophila* survives in nature by virtue of a differentiation cycle in which distinct cell types interconvert in response to environmental and metabolic fluctuations. In its planktonic transmissive form, *L. pneumophila* is motile, resistant to multiple environmental stresses, including nutrient starvation, and infectious to host cells (Rowbotham, 1983, 1986; Byrne and Swanson, 1998). In the transmissive phase, effectors translocated across the bacterial membrane and virulence factors on the *L. pneumophila* surface arrest phagosome maturation to establish a replication vacuole derived from the host's endoplasmic reticulum (Swanson and Isberg, 1995; Byrne and Swanson, 1998; Joshi et al., 2001; Fernandez-Moreira et al., 2006; Ensminger and Isberg, 2009; Isberg et al., 2009; Rolando and Buchrieser, 2012; Amyot et al., 2013; Figure 1). The replicative form of *L. pneumophila* multiplies intracellularly within such vacuoles, which in some host cells mature into acidic lysosomal vacuoles that support bacterial growth (Sturgill-Koszycki and Swanson, 2000; Xu et al., 2010).

*L. pneumophila* can also differentiate to other cell types that remain to be characterized in molecular detail. During its life cycle inside protozoa, HeLa cells, epithelial cells or even clinical specimens, transmissive *L. pneumophila* differentiate further to a mature intracellular form (MIF), a highly infectious, metabolically-resting, cyst-like form that is observed late during infection (Faulkner and Garduno, 2002; Garduno et al., 2002; Greub and Raoult, 2003; Faulkner et al., 2008; Garduno, 2008). The resilience of extracellular *L. pneumophila* is further evidenced by its survival in a viable but non-culturable (VBNC) state that can arise when either transmissive or stationary phase *L. pneumophila* or MIFs are exposed to severe conditions in water environments (Steinert et al., 1997; Garduno, 2008; Al-Bana et al., 2014). *L. pneumophila* can also form monospecies biofilms or colonize multi-species biofilm communities (Abdel-Nour et al., 2013).

The ability to exploit extracellular and intracellular niches and endure environmental stresses, including nutritional starvation, equips *L.pneumophila* to persist in nature. Indeed, adaptation to the highly variable conditions encountered by *L.pneumophila* requires swift morphogenetic and physiological transformations (Byrne and Swanson, 1998; Garduno et al., 2002; Molofsky and Swanson, 2004; Garduno, 2008). Accordingly, the *L. pneumophila* life cycle is controlled by multipronged regulatory systems that control gene expression; these include a variety of transcriptional regulatory proteins, two-component systems, non-coding RNA (ncRNA) molecules, the stringent response pathway, and metabolites (Hammer and Swanson, 1999; Bachman and Swanson, 2001, 2004; Hammer et al., 2002; Molofsky and Swanson, 2003; Molofsky et al., 2005; Hovel-Miner et al., 2009; Dalebroux et al., 2009, 2010; Edwards et al., 2009, 2010; Sahr et al., 2009; Albert-Weissenberger et al., 2010).

Whether in extracellular or intracellular environments, differentiation of transmissive *L. pneumophila* to the replicative form is tightly coupled to its metabolic state (Hammer and Swanson, 1999; Sauer et al., 2005; Dalebroux et al., 2009; Edwards et al., 2009, 2010). When transmissive *L. pneumophila* encounter abundant nutrients in their environment, such as amino acids, these metabolites trigger differentiation to the replicative form (Hammer and Swanson, 1999; Sauer et al., 2005; Figure 1). As *L. pneumophila* multiplies, nutrient consumption or accumulation of certain metabolites alters the bacterium's nutritional milieu. The metabolic changes that these alterations provoke are relayed to regulatory components that include the stringent response, the LetA/S two-component system and alternative sigma factors to trigger differentiation of *L. pneumophila* into the transmissive form (Edwards et al., 2009, 2010; Figure 1). The transmissive form of *L. pneumophila* is suited to seek a new niche that supports replication. Thus, metabolic cues serve not only as gauges of fitness of the environment for replication but also as triggers of differentiation into other cell types, such as the transmissive form.

The sensing of metabolic signals to adopt a particular cell type does not constitute the only strategy intracellular *L. pneumophila* utilizes to meet its physiological and metabolic needs. Among the many effector molecules translocated by the Icm/Dot type IVB secretion system are proteins that exploit host pathways either by altering eukaryotic phospholipid metabolism or by utilizing the host proteasome to generate an amino acid supply (Al-Quadan et al., 2012; Viner et al., 2012; Bruckert et al., 2014). As another means to exploit the host environment for replication, intracellular *L. pneumophila* employs components of its own metabolism such as nutrient salvaging systems (Cianciotto et al., 2005; Sauer et al., 2005; Yip et al., 2011; Fonseca et al., 2014). In this review, we showcase the growing body of knowledge of the metabolic components derived from both bacterial and host sources that contribute to *L. pneumophila* replication within host cells.

## *In vitro* experimental probes of intracellular metabolism by *Legionella pneumophila*

The study of *L. pneumophila* metabolism, particularly of intracellular forms, has been expedited by a number of genetic and molecular tools and *in vitro* studies of metabolism. Of particular importance has been the ability to generate mutants using various methodologies, including enrichments (Berger and Isberg, 1993; Swanson and Isberg, 1996), transposon libraries (Hammer and Swanson, 1999; Sauer et al., 2005), natural competence (Sexton and Vogel, 2004; Buchrieser and Charpentier, 2013), and recombineering (Bryan et al., 2011). Using these techniques, *L. pneumophila* mutants defective at particular stages of the intracellular life cycle have been isolated and characterized to gain insight into both the fate of *L. pneumophila* in phagocytes and the host components that support infection.

The ability to study the growth-phase dependent expression of panels of replicative and transmissive traits of *L. pneumophila* in synchronous broth cultures has also greatly advanced our understanding of the regulation of this bacterium's life cycle and its metabolism (Rowbotham, 1986; Byrne and Swanson, 1998; Molofsky and Swanson, 2004; Dalebroux et al., 2009; Edwards et al., 2009). This knowledge has enhanced our ability to assess the requirement of specific functions for particular cell types *in vivo* (Sauer et al., 2005; Dalebroux et al., 2010). For instance, *L. pneumophila* mutants of the phagosomal transporter A (*phtA*) display an intracellular growth defect that can be bypassed *in vivo* by threonine supplementation (Sauer et al., 2005). Moreover, without threonine supplementation, *phtA* mutants are locked in the transmissive form and fail to differentiate into the replicative form inside macrophages. Similarly, the reciprocal expression of replicative and transmissive traits can be utilized to assess the requirement of various gene products for bacterial differentiation in the host (Hammer et al., 2002; Molofsky and Swanson, 2003; Dalebroux et al., 2010).

The response of *L. pneumophila* to particular metabolic cues has been probed by coupling various molecular methods to assays of growth-phase dependent traits (Hammer and Swanson, 1999; Edwards et al., 2009). These include the use of transcriptional fusions (Hammer and Swanson, 1999; Hammer et al., 2002; Edwards et al., 2009), phenotype microarrays (Sauer et al., 2005; Edwards et al., 2009) and transcriptional profile studies (Bruggemann et al., 2006; Dalebroux et al., 2010; Edwards et al., 2010; Hovel-Miner et al., 2010; Faucher et al., 2011). These tools have facilitated experiments to probe how intracellular *L. pneumophila* sense its nutritional milieu in macrophages and adopt the appropriate cell fate (Dalebroux et al., 2009, 2010).

Insights into the nutritional requirements of *L. pneumophila* in host cells have also been obtained from classical nutritional studies using broth cultures. Groundbreaking experiments revealed key nutritional requirements of *L. pneumophila*: the absolute requirement for oxygen for its metabolism, the use of amino acids as major sources of carbon and energy (Pine et al., 1979; Reeves et al., 1981; Ristroph et al., 1981; Tesh et al., 1983), the requirements for specific nutrients in host cells (Sauer et al., 2005), the utilization of host-derived nutrients (Al-Quadan et al., 2012; Bruckert et al., 2014), and the identification of nutritional auxotrophies (Pine et al., 1979; George et al., 1980; Ristroph et al., 1981; Tesh and Miller, 1981). In other instances, the ability to assess the nutritional requirements of *L. pneumophila* in broth has allowed genes of unknown function to be linked to particular metabolic processes (Sauer et al., 2005; Fonseca et al., 2014).

Heterologous genetic systems have enabled the function of several *L. pneumophila* proteins to be deduced. This approach proved instrumental in gathering supporting evidence for *in silico* analyses and observed intracellular growth phenotypes (Molofsky and Swanson, 2003; Bachman and Swanson, 2004; Nasrallah et al., 2011; Viner et al., 2012; Fonseca et al., 2014).

Isotopologue profiling has been implemented as a tool to probe the metabolic pathways of assimilation of various precursors into building blocks in *L. pneumophila* (Eylert et al., 2010). First, the *in vitro* isotopologue studies confirmed several of the amino acid auxotrophies that had been observed in broth cultures of *L. pneumophila*, namely those of methionine, arginine, valine, leucine, and isoleucine. Furthermore, by using isotopologues of glucose and the amino acid serine, the investigators determined that *L. pneumophila* can metabolize glucose using the Entner-Doudoroff (ED) pathway and that carbon from glucose is primarily used for the synthesis of poly-3-hydroxybutyrate, a storage compound that *L. pneumophila* utilizes under nutrient starvation conditions (James et al., 1999; Garduno et al., 2002). The contribution of the ED pathway was tested by generation of a *L. pneumophila* strain Paris mutant in the putative glucose-6-phosphate-1-dehydrogenase gene (*zwf*) (Eylert et al., 2010). In multiple-round co-infection experiments, the *zwf* mutant was outcompeted by wild type after >2 weeks, pointing to a contribution of the *zwf* gene in fitness during prolonged co-culture with amoebae. How the *zwf* gene in particular and the ED pathway in general contribute to *Legionella* fitness in the environment requires further investigation. Thus, implementation of isotopologue labeling techniques has increased our understanding of *L. pneumophila* metabolism and has led to further development of this methodology to study the intracellular metabolism of *L. pneumophila* (Heuner and Eisenreich, 2013).

Our current knowledge of *L. pneumophila* metabolism relies on the integration of various *in vitro* and *in vivo* approaches. A caveat of incorporating the results of various experimental approaches to understanding *L. pneumophila* physiology is that the metabolic state of the bacterium may vary widely depending on the cell type, growth culture conditions, and sensitivity of the assays employed, which may lead to apparent discrepancies in experimental results.

## Regulation of the *L. pneumophila* life cycle by metabolites

A salient feature of bacterial cells is their ability to adapt to constant physicochemical changes in their environment. The tuning of bacterial physiology by controlling gene expression at the transcriptional and translational level has been the object of intense study (Henkin, 2000; Browning and Busby, 2004; Serganov and Patel, 2012). In addition to gene regulation, bacteria ensure energy efficiency and persistence in nature by employing another strategy, one perhaps less appreciated by molecular biologists: metabolic control. The latter mainly consists of post-translational control of metabolism by metabolites. Mechanisms of post-translational control common in biology include allosteric control and feedback inhibition (Sanwall, 1970; LaRossa et al., 1987; Allen et al., 2002; Starai and Escalante-Semerena, 2004; Wolfe, 2005; Bazurto and Downs, 2014). The overlap between many of these genetic and metabolic regulatory machineries further supports how bacterial physiology is subject to sophisticated control. The collective contributions of these studies have been invaluable in our current understanding of the factors that allow bacterial adaptation to a wide variety of alterations in their surroundings.

That the *L. pneumophila* intracellular life cycle is also subject to regulation by metabolites is a fact that is supported by numerous lines of evidence (Byrne and Swanson, 1998; Hammer and Swanson, 1999; Sauer et al., 2005; Dalebroux et al., 2009; Edwards et al., 2009; Hovel-Miner et al., 2010). In several examples we shall discuss here, it is clear that a subset of metabolites exert their effect, at least in part, at the transcriptional level. Whether various metabolites govern the *L. pneumophila* life cycle by mechanisms such as allostery is an intriguing question that requires further experimentation, particularly, biochemical analyses.

### Amino acids

The preference of *L. pneumophila* for amino acids as main sources of carbon and energy was first deduced from experiments in broth cultures (Pine et al., 1979; Reeves et al., 1981; Ristroph et al., 1981; Tesh et al., 1983). More recently, isotopologue studies further indicated that the amino acid serine supports carbon metabolism in *L. pneumophila* as well as confirmed some of the amino acid auxotrophies that had been determined previously using broth cultures (Pine et al., 1979; George et al., 1980; Ristroph et al., 1981; Tesh and Miller, 1981; Eylert et al., 2010). These and other amino acid auxotrophies had also been suggested by *in silico* genomic analyses (Fonseca et al., 2008). Moreover, as exemplified by the characterization of *phtA* mutants in macrophages, differentiation of transmissive *L. pneumophila* to the replicative form responds to cues that indicate amino acid abundance. Thus, a plentiful amino acid supply promotes replication of either intracellular or extracellular *L. pneumophila*.

Fundamental to our current understanding of the *L. pneumophila* life cycle has been the observation that, when starved for amino acids, *L. pneumophila* switches from the replicative form to the transmissive form. Transmissive *L. pneumophila* express a number of virulence traits such as motility, lysosome evasion and cytotoxicity (Byrne and Swanson, 1998; Hammer and Swanson, 1999; Bachman and Swanson, 2001; Lynch et al., 2003) that promote escape from a spent host and infection of a naïve one. The response to amino acid starvation is coordinated by synthesis of the alarmone ppGpp by the RelA synthetase (Hammer and Swanson, 1999; Dalebroux et al., 2009, 2010). The ppGpp signal transducer in turn induces expression of a series of genes typical of stress responses in bacteria (Gentry et al., 1993; Durfee et al., 2008; Traxler et al., 2008; Zhao and Houry, 2010). Indeed, ppGpp triggers activation of various regulatory components in *L. pneumophila*, such as alternative sigma factors (Rasis and Segal, 2009; Sahr et al., 2009; Edwards et al., 2010), in a complex regulatory cascade that also requires the LetA/LetS two-component system (Hammer et al., 2002; Dalebroux et al., 2010). The dramatic morphogenetic and physiological alterations exhibited by *L. pneumophila* in response to amino acid scarcity or abundance underscore the importance of these nutrients to its life cycle and virulence.

The ability of amino acids to interact with regulatory components that are pivotal for intracellular replication by *L. pneumophila* was further evidenced in a study of arginine metabolism in this bacterium (Hovel-Miner et al., 2010). The study stemmed from the experimental observation that mutations in the arginine repressor (ArgR) protein of *L. pneumophila* confer an intracellular replication defect within host *Acanthamoeba castellanii* (Hovel-Miner et al., 2009). ArgR protein represses genes required for *de novo* arginine biosynthesis from glutamate (Maas, 1994). Whereas *L. pneumophila* lacks many of the genes that other species require for early steps in arginine biosynthesis, the bacterium can synthesize arginine in defined medium supplemented with either of the precursors ornithine or citrulline (Tesh et al., 1983; Hovel-Miner et al., 2010). Hovel-Miner and colleagues demonstrated that *L. pneumophila* lacking the genes for either a putative amino acid ABC transporter or the last step of the arginine biosynthetic route fail to grow in defined medium unless provided with arginine. Nevertheless, both mutant strains do grow intracellularly, and their growth was not stimulated by addition of arginine to the infection buffer. Accordingly, it was deduced that *L. pneumophila* can tolerate arginine auxotrophy because the pathogen obtains arginine from the host to support intracellular replication. The latter finding was intriguing considered in the context of the intracellular growth defect conferred by *argR* mutants in amoeba. Global transcriptional analyses employed to address this paradox revealed that in *L. pneumophila* the ArgR repressor regulates a number of components apparently unrelated to arginine metabolism but required for intracellular replication.

Among the components regulated by ArgR in *L. pneumophila* are genes encoding proteins of the Icm/Dot Type IV secretion system (subject to negative regulation) and its translocated substrates (subject to positive regulation). These data led to the proposal that arginine and ArgR are required for assembly of the Icm/Dot system at the appropriate time during the *L. pneumophila* life cycle and for the synthesis of functions that promote establishment of the *L. pneumophila* replication vacuole.

Noteworthy is the fact that in organisms like *Escherichia coli* ArgR exerts its regulatory effect with arginine as a co-repressor (Lim et al., 1987; Maas, 1994). The results of the global transcriptional analyses suggest that arginine has a similar role in *L. pneumophila* (Hovel-Miner et al., 2010). This finding together with the similarity of amino acid sequences and DNA-binding sites of the ArgR protein in *L. pneumophila* to ArgR proteins of *E. coli* and *Bacillus subtilis* further highlight the importance of amino acid metabolic cues to the regulatory circuits that control intracellular replication and differentiation in *L. pneumophila*.

### Fatty acids

Short-chain fatty acids (SCFAs) are known regulators of metabolism and virulence in bacteria. For example, in *Salmonella enterica* serovar Typhimurium, exposure to SCFAs like acetate and propionate can have deleterious effects on cell metabolism. Among these are effects on acyl-CoA homeostasis, depletion of free CoA pools, and accumulation of toxic metabolic intermediates, which have also been observed in other bacteria like *E. coli* and *Rhodobacter sphaeroides* (Maruyama and Kitamura, 1985; Man et al., 1995; Horswill et al., 2001; Starai et al., 2003; Starai and Escalante-Semerena, 2004; Garrity et al., 2007). SCFAs are present at high concentrations in the human gastrointestinal tract (Cummings et al., 1987) and stimulate invasion of the intestinal epithelium by *S. enterica* (Lawhon et al., 2002; Huang et al., 2008; Hung et al., 2013).

Analogously, the SCFAs formate, acetate, propionate and butyrate are among the metabolic signals that elicit differentiation of replicative *L. pneumophila* to the transmissive form, as revealed by phenotypic microarray analysis (Edwards et al., 2009). At concentrations of 10 mM, SCFAs halt *L. pneumophila* growth without affecting cell viability and induce expression of motility, cytotoxicity, sodium sensitivity and avoidance of lysosomal degradation, all hallmark traits of transmissive *L. pneumophila*. The LetA/LetS two-component system is pivotal to the response of *L. pneumophila* to SCFAs as metabolic cues, as *letA* or *letS* mutants are blind to SCFA signals for differentiation. The morphogenetic alterations observed are not merely due to changes in pH, as inorganic acids like hydrochloric acid, even in excess of 10 mM, fail to elicit differentiation of *L. pneumophila in vitro*.

A formal possibility was that, to coordinate the response to SCFAs, *L. pneumophila* employs the acetate kinase (Ack)/phosphotransacetylase (Pta) system that converts SCFAs like acetate into their acyl-phosphate (P) or acyl-coenzyme A (CoA) derivatives. This system is utilized by bacteria like *E. coli*, *S. enterica* and *L. monocytogenes* to control several cellular processes (Starai and Escalante-Semerena, 2004; Wolfe, 2005; Gueriri et al., 2008). In this two-enzyme system, acetate may be phosphorylated by Ack with subsequent conversion to acetyl-CoA by Pta. The reactions catalyzed by Ack or Pta can be reversed according to cell needs. The Ack and Pta enzymes may be used by bacteria in several ways: (i) to generate acetyl-P, a high energy intermediate that may serve as a signal for two-component systems involved in activities like biofilm formation, flagellar biosynthesis, motility and pathogenesis (Wolfe et al., 2003; Wolfe, 2005, 2010; Gueriri et al., 2008; Sze and Li, 2011), (ii) to assimilate acetate into acetyl-CoA, or (iii) to dissimilate acetyl-CoA into acetate (Starai and Escalante-Semerena, 2004; Wolfe, 2005).

Unlike in other systems, *L. pneumophila* does not appear to assimilate an excess of these SCFAs through the proteins encoded by *ackA2* and *pta* to relay metabolic signals to the LetA/LetS two-component system. In particular, *L. pneumophila* strains mutated in the genes proposed to encode Ack and Pta nevertheless differentiate from the replicative to the transmissive form in the presence of excess acetate or propionate (Edwards et al., 2009). Nonetheless, SCFAs do provoke the regulatory cascade that leads to differentiation of replicative *L. pneumophila* to the transmissive form using the stringent response via the bifunctional ppGpp synthetase/hydrolase SpoT enzyme (Dalebroux et al., 2009). It cannot be ruled out, however, that apart from the stringent response, *L. pneumophila* may use pathways distinct to those described for other intracellular pathogens to respond to alterations in fatty acid metabolism and maintain CoA homeostasis when exposed to excess SCFAs.

An additional feature of the integrated nature of metabolism and differentiation in *L. pneumophila* is that the activities of the RelA and SpoT enzymes are dedicated to respond to distinct metabolic signals. Under conditions of fatty acid metabolic stress, ppGpp is synthesized by the bifunctional ppGpp synthetase/hydrolase (SpoT) enzyme. Conversely, RelA is employed by *L. pneumophila* to mount a response to amino acid starvation signals (Dalebroux et al., 2009; Edwards et al., 2009). Intracellular *L. pneumophila* also exhibit distinct requirements for RelA and SpoT as the ppGpp synthetase activity encoded by *relA* is dispensable for intracellular replication, but the ppGpp synthetase/hydrolase activities encoded by the *spoT* gene are not (Dalebroux et al., 2009).

Whereas ppGpp synthesis is required for *L. pneumophila* to express the virulence factors that are required for infection and establishment of the compartment in which it will replicate, ppGpp hydrolysis by SpoT is required for intracellular bacteria to initiate replication (Dalebroux et al., 2009). In fact, *L. pneumophila relA spoT* mutant strains, blocked in ppGpp synthesis, exhibit poor infectivity to macrophages and the bacteria that survive after initial infection are degraded as they are unable to avoid phagosome-lysosome fusion. Experiments in which ppGpp synthetase activity was supplied to *relA spoT* mutant strains by providing *relA*^+^ or *spoT*^+^ alleles in multicopy indicated that the ppGpp required for infection of the host may be synthesized by either RelA or SpoT. However, the ppGpp synthetase activity of SpoT is specifically required for secondary infections. These data established that *L. pneumophila* requires careful regulation of ppGpp synthesis and hydrolysis to respond to metabolic signals and differentiate accordingly.

When exposed to excess SCFAs, *L. pneumophila relA spoT* mutant strains fail to be cytotoxic to macrophages. Furthermore, *L. pneumophila* strains carrying a particular allele of the *spoT* gene (*spoT-A413E*) in multicopy were blind to perturbations in fatty acid metabolism as they exhibited enhanced cytotoxicity regardless of SCFA exposure (Dalebroux et al., 2009). It was postulated that the increased cytotoxicity phenotype was due to two key features of the mutant SpoT enzyme: increased ppGpp synthetase activity and inability to interact with the fatty acid metabolism machinery of the cell. This line of experiments further demonstrated that the balance between ppGpp synthesis and hydrolysis is critical to the *L. pneumophila* intracellular lifestyle.

In *E. coli*, SpoT activity is modulated via direct interactions with the acyl-carrier protein (ACP) (Battesti and Bouveret, 2006; Angelini et al., 2012), and molecular genetic analyses indicate this is also the case for *L. pneumophila* (Dalebroux et al., 2009). ACP is a crucial component of the fatty acid and phospholipid biosynthetic machinery in bacteria and eukaryotes (Lombard et al., 2012) that is responsible for carrying fatty acid chains as acyl derivatives attached to a 4′-phosphopantetheine prosthetic group in ACP. Indeed, the interaction of SpoT with ACP requires that the phosphopantetheine moiety be attached to ACP, indicating that SpoT interacts with functional ACP (Battesti and Bouveret, 2006). The structure of ACP is altered by perturbations in fatty acid metabolism, such as inhibition of fatty acid synthesis caused by mutations in fatty acid biosynthetic enzymes (Jackowski and Rock, 1983, 1987). Thus, the conformation of ACP reflects the status of fatty acid metabolism, which it then relays to the SpoT enzyme. SpoT in turn responds by either hydrolyzing ppGpp when fatty acid biosynthesis is uncompromised or synthesizing the alarmone when fatty acid metabolism is perturbed.

*L. pneumophila* also appears to integrate fatty acid metabolism with the expression of virulence factors through alterations in acyl-ACP forms (Edwards et al., 2009). The *L. pneumophila* Philadelphia-1 genome contains several orthologs of genes encoding acetyl-CoA carboxylase (ACC), β-ketoacyl-ACP synthases I (FabB), and II (FabF) (Edwards et al., 2009). When *L. pneumophila* is exposed to known inhibitors of the above mentioned gene products, individually, as in the case of the antibiotic cerulenin, or in combination, it differentiates to the transmissive form. Differentiation under these conditions is proposed to occur by accumulation of fatty acid metabolites that are purported to stall fatty acid metabolism and alter the conformation of ACP. Exposure to SCFAs may also mimic this metabolic state. Analysis of acyl-ACP species of *L. pneumophila* cells that have been exposed to SCFAs lend support to this idea: the acyl-ACP profiles of exponentially replicating bacteria exposed to acetate or propionate mimic those in of cells in the transmissive state (Edwards et al., 2009).

Regardless of whether fatty acid metabolism is altered by pharmacological inhibition of enzyme activity or the presence of particular fatty acid metabolites, both the stringent response commanded by the SpoT enzyme and the LetA/LetS two-component system are required components of the multilayered regulatory strategy employed by *L. pneumophila* to monitor the state of fatty acid metabolism (Dalebroux et al., 2009; Edwards et al., 2009).

### Nicotinic acid

The metabolism of NAD^+^ and NADP^+^ is necessary for redox reactions of catabolism and anabolism in living organisms. In addition, NAD^+^ is a required substrate in reactions of DNA synthesis, posttranslational modification of proteins, and cell signaling processes (reviewed in Gazzaniga et al. 2009). The pyridine derivative nicotinic acid is a known component of NAD^+^ salvage pathways. Nicotinic acid may be produced from NAD^+^ degradation in cells and can be recycled back to NAD^+^ via the pyridine nucleotide cycle salvage pathway, also known as the Preiss-Handler pathway (Foster et al., 1979; Foster and Baskowsky-Foster, 1980; Hillyard et al., 1981; Gazzaniga et al., 2009; Houtkooper et al., 2010; Keseler et al., 2011). Exogenous nicotinic acid can also be assimilated via the same route.

Whereas bacteria like *E. coli* and *S. enterica* are capable of *de novo* NAD^+^ biosynthesis, microbes like *Bordetella pertussis* and *Candida glabrata* are not (Domergue et al., 2005; Ma et al., 2007; Gazzaniga et al., 2009). Moreover, the latter organisms can salvage nicotinic acid to satisfy their NAD^+^ requirement (McPheat et al., 1983; Domergue et al., 2005; Ma et al., 2007). *B. pertussis* and *C. glabrata* are human pathogens in which nicotinic acid metabolism also regulates the expression of virulence factors such as adhesins, toxins, and agglutinating factors (Schneider and Parker, 1982; McPheat et al., 1983; Cotter and DiRita, 2000; Domergue et al., 2005).

In *B. pertussis*, nicotinic acid modulates the activity of the BvgA/BvgS two-component system, a master controller of a spectrum of phenotypic states of *B. pertussis* known as Bvg phases (Melton and Weiss, 1989; Cummings et al., 2006). For example, during the Bvg^+^ phase, *B. pertussis* is equipped to colonize the human respiratory tract, whereas in the intermediate Bvg^i^ phase, *B. pertussis* is more suited for transmission (Vergara-Irigaray et al., 2005). The BvgA/BvgS two-component system works through a four-step phosphorelay that is singular among two-component systems, not only due to the additional steps of the phosphorelay, but also because three of the four phosphorelay domains are found within the BvgS protein itself rather than in three different proteins, as in the *Bacillus subtilis* sporulation system (Appleby et al., 1996). By mechanisms yet unknown, nicotinic acid inactivates the BvgA/BvgS two-component system, thus repressing expression of virulence factors (Schneider and Parker, 1982; McPheat et al., 1983; Cotter and DiRita, 2000; Cummings et al., 2006). The BvgA/S systems controls panels of traits whose expression depends on the concentration of BvgA-phosphate (Bvg~P), thereby generating a variety of phenotypic states for *B. pertussis* bacteria (Williams and Cotter, 2007; Boulanger et al., 2013).

The LetA/LetS two-component system in *L. pneumophila* has homology to the *B. pertussis* BvgA/BvgS system (Edwards et al., 2010), particularly in the polydomain structure of the LetS protein. The sequence similarities of the two systems and the fact that the LetA/LetS system controls a panel of transmissive traits in *L. pneumophila* led to the idea that nicotinic acid might also modulate gene expression in this bacterium.

A high concentration of nicotinic acid (5 mM) halts growth of replicative *L. pneumophila* and triggers premature expression of transmissive traits, such as motility, evasion of lysosomal degradation, sodium sensitivity, and cytotoxicity to macrophages (Edwards et al., 2013). The LetA/LetS system is required for full induction of the flagellin promoter, but is not required for nicotinic acid to exert its inhibitory effect on growth.

Global transcriptional analyses of replicative *L. pneumophila* exposed to nicotinic acid revealed that this metabolite triggers expression of genes typical of the transmissive state of the bacterium, including those encoding: (i) virulence factors like the *ralF* gene and components of the Dot/Icm apparatus; (ii) the flagellar regulon, and (iii) alternative sigma factors, like *rpoN, rpoH*, and *fliA*. Moreover, the transcriptome analyses identified a locus whose expression is altered as a specific response to nicotinic acid exposure. In particular, open-reading frames (ORFs) *lpg0272* and *lpg0273* were induced 9-fold and 35-fold by treatment with nicotinic acid. ORF *lpg0272* is annotated as a cysteine transferase activity, and ORF *lpg0273* is predicted to encode a protein of the major facilitator superfamily (MFS) of transporters. In particular, the predicted protein structure of Lpg0273 resembles the EmrD multidrug efflux pump of *E. coli* (Yin et al., 2006; Edwards et al., 2013). Interestingly, two ORFs whose products are proposed to be involved in NAD^+^ salvage are encoded immediately upstream of the *lpg0272-3* locus (Caspi et al., 2012; Edwards et al., 2013). Nevertheless, their expression levels were unchanged in response to nicotinic acid (Edwards et al., 2013).

Additional genetic analyses revealed that the product of ORF *lpg0273* equips *L. pneumophila* to cope with the stress of exposure to high concentrations of nicotinic acid. *L. pneumophila* strains that harbor a deletion of the *lpg0273* ORF exhibit increased sensitivity to nicotinic acid. Conversely, strains carrying *lpg0273* in multicopy are more resistance to nicotinic acid (Edwards et al., 2013). Given the properties conferred by *lpg0273* to *L. pneumophila* exposed to nicotinic acid, ORF was designated *mnrA* for “MFS nicotinic acid responder” (Edwards et al., 2013).

The specific mechanisms by which nicotinic acid impacts metabolism and virulence gene expression in *L. pneumophila* and other bacteria still remain to be determined. The transcriptional profiles of transmissive bacteria and replicative bacteria exposed to nicotinic acid harbor several clues that promise to unveil the molecular and metabolic response of *L. pneumophila* to the pyridine compound. First, the loci that displayed the greatest transcriptional changes in response to nicotinic acid are near loci that are proposed to encode NAD^+^ salvage functions. Second, one of the ORFs that is highly expressed under nicotinic acid stress confers *L. pneumophila* with protection from this metabolite. This transporter may prove analogous to transporters of *Saccharomyces cerevisiae* that balance import and export of NAD^+^ precursors like nicotinamide riboside and nicotinic acid to support NAD^+^homeostasis (Belenky et al., 2011). Third, ORF *lpg0274*, proposed to encode a transcriptional regulator of the LysR family, is positioned 3′ of ORF *lpg0273* and is also highly transcribed in the presence of nicotinic acid. This ORF may prove to be a regulator of nicotinic acid metabolism and of the nearby NAD^+^ salvage loci in *L. pneumophila*. The coordinated response of *L. pneumophila* to metabolites like nicotinic acid is another display of the strategies this microbe employs to respond to metabolic changes.

### Other metabolites

Several other metabolites were discovered to trigger premature differentiation of *L. pneumophila*, including, but not limited to, deoxyadenosine, deoxyribose, hydroxylamine, and 4-hydroxybenzoic acid (Edwards et al., 2009). Whether these compounds exert their effect by impacting additional metabolic routes requires additional genetic and biochemical analyses.

## Bacterial metabolic components required for virulence and intracellular growth

### Phagosomal transporters (Phts) as nutrient salvaging systems for intracellular *L. pneumophila*

The *L. pneumophila* phagosomal (Pht) transporter sub-family of the major facilitator superfamily (MFS) of transporters in Chen et al. (2008) was uncovered through genetic characterization of the *phtA* locus, which is adjacent to *letE*, a locus that encodes an enhancer protein of transmissive trait expression (Bachman and Swanson, 2004). *L. pneumophila phtA* mutants exhibit a marked intracellular growth defect (Sauer et al., 2005). An interesting feature of *phtA* mutants is that they efficiently infect host cells and are able to establish an ER-derived compartment like wild-type *L. pneumophila* (Sauer et al., 2005). However, instead of multiplying like wild-type bacteria, *phtA* mutants remain as single cells in the vacuolar compartment and continue to express transmissive traits (Sauer et al., 2005). Thus, the intracellular growth defect of *phtA* mutants is due to the inability of the bacterial cells to differentiate from the transmissive to the replicative form. The differentiation phenotype of *phtA* mutants can be corrected by addition of threonine, threonine-containing peptides or amino acid supplements, or by supplying the wild-type *phtA* allele *in trans* (Sauer et al., 2005). Given the homology of *phtA* to genes encoding MFS transporters, it was deduced that PhtA affords a threonine acquisition function that equips *L. pneumophila* to differentiate and replicate in macrophages.

Additional molecular, genetic and *in silico* analyses revealed the presence of *phtA* homologues within the *L. pneumophila* Philadelphia-1 genome and within intracellular microbes like *Francicella tularensis* and *Coxiella burnetti* (Sauer et al., 2005; Chen et al., 2008). However, the *L. pneumophila* genome encodes the highest number of Pht functions (Chen et al., 2008).

In addition to *phtA*, 5 additional *pht* loci have been identified as necessary for intracellular replication, namely, *phtC, phtD, phtE, phtF*, and *phtJ* (Sauer, 2006; Fonseca et al., 2014). Whereas, the *phtJ* gene product is required for valine acquisition (Sauer, 2006), the products of the *phtC-D* locus are involved in thymidine salvage in *L. pneumophila* (Fonseca et al., 2014). Thus, *pht* loci are devoted not only to the assimilation of amino acids, but also to additional metabolic functions. The role of the *phtE* and *phtF* loci which are adjacent in the *L. pneumophila* genome remains unknown.

The link of the *phtC-D* locus to thymidine salvage in *L. pneumophila* was deduced by analysis of the *L. pneumophila* genome and phenotypic studies that have been recently reported (Fonseca et al., 2014). The *phtC* and *phtD* genes are located in tandem in the *L. pneumophila* genome with the *tdk* gene, proposed to encode thymidine kinase and immediately upstream of *phtC*, and the *deoB* gene proposed to encode phosphopentomutase and immediately downstream of *phtD*. Since Tdk and DeoB proteins are well-documented thymidine salvage functions in bacteria like *E. coli*, the potential involvement of the *L. pneumophila phtC-D* locus in thymidine salvage was investigated. To assess the role of the *phtC* and *D* gene products in thymidine salvage, we exploited the thymidine auxotrophy of the *L. pneumophila* Lp02 parent strain, conferred by a mutation in *thyA*, encoding the putative thymidylate (dTMP) synthetase enzyme (Berger and Isberg, 1993). Indeed, *phtC* and *phtD* mutants exhibited distinct, but sharp phenotypes when exposed to thymidine starvation conditions *in vitro* (Fonseca et al., 2014). When deprived of exogenous thymidine, *L. pneumophila thyA* strains are highly resilient to starvation conditions, a trait that is surpassed by *thyA phtD* mutant strains. In stark contrast, *thyA phtC* mutant strains not only fail to grow, but quickly lose viability. The thymidine starvation phenotypes of the *thyA phtC* mutant strains are corrected by addition of excess exogenous thymidine or by a *phtC*^+^ allele in multicopy.

The sequence homology of PhtC to that of MFS transporters suggests that this protein may transport exogenous thymidine. In fact, *L. pneumophila phtC*^+^ in multicopy supplies pyrimidine uptake to an *E. coli* strain that lacks all nucleoside transport systems known for that organism, but *phtD*^+^ does not (Fonseca et al., 2014).

The role of the *phtC-D* locus in assimilation of exogenous thymidine in *L. pneumophila* was further tested in isogenic *thyA*^+^ strains. The ability of the pyrimidine nucleoside analog 5-fluorodeoxyuridine (FUdR) to block *de novo* dTMP synthesis and thus cell growth was exploited in phenotypic assays of *thyA*^+^ *phtC* and *thyA*^+^ *phtD* strains. It was initially hypothesized that *phtC* or *phtD* mutant strains, lacking nucleoside uptake functions, would be resistant to growth inhibition by the analog. Surprisingly, the *L. pneumophila thyA*^+^ parent is highly resilient to FUdR inhibition, but sensitivity to the analog is increased by *phtC* or *phtD* mutations. In particular, a *phtC* mutation results in full inhibition of growth by FUdR. Again, the phenotypes observed are striking, as impairment of nucleoside transport would result in decreased ability of the analog to enter the cell. FudR inhibition of *L. pneumophila thyA*^+^ *phtC* and *thyA*^+^ *phtD* strains is corrected by addition of exogenous thymidine and/or *phtC*^+^ in multicopy. These results led to the notion that the products of the *phtC-D* locus affect dTMP synthesis by mechanisms that extend beyond nucleoside salvage and that impact *de novo* routes in as yet undefined ways.

The *thyA phtC* and *thyA phtD* growth defects in macrophages were not correctible by addition of exogenous thymidine, restoration of thymidine prototrophy to the mutant strains, or addition of each loci in various combinations (Fonseca et al., 2014). Instead, the whole *tdk-phtC-phtD-deoB* locus was required to complement the intracellular growth defect in the macrophage host. Consistent with this result, mRNA analysis of wild-type *L. pneumophila* grown in the presence of thymidine reveals that under these conditions, *tdk, phtC, phtD*, and *deoB* are co-transcribed. Thus, whereas under defined *in vitro* conditions *phtC*^+^ in multicopy or exogenous thymidine are sufficient to correct the defects of mutants in the *phtC-D* locus, intracellular growth demands more stringent conditions. The distinct and sometimes contrasting thymidine salvage phenotypes exhibited by *phtC* and *phtD* mutant strains suggest that nucleoside metabolism, like in other bacteria, is subject to complex transcriptional and metabolic regulation. Additional molecular and biochemical analyses are required to ascertain the mechanisms by which the *phtC-D* locus contributes to thymidine salvage in *L. pneumophila* and to its intracellular life cycle.

### Iron metabolism and cytochromes

Like for most living organisms, iron is an essential nutrient for *L. pneumophila* (Reeves et al., 1981). Iron is a constituent of prosthetic groups like heme and iron-sulfur clusters used by several enzymes with diverse and central roles in metabolism such as electron transfer reactions, substrate binding and catalysis, and oxygen transport. The major iron-containing protein in *L. pneumophila* is the homolog to *E. coli* aconitase enzyme of the tricarboxylic acid cycle (Mengaud and Horwitz, 1993). In addition, the genomes of several *L. pneumophila* strains contain genes proposed to encode iron-sulfur proteins and heme-containing cytochrome *b* and *c*-type cytochromes (Cazalet et al., 2004; Chien et al., 2004; D'Auria et al., 2010; Caspi et al., 2012).

Iron also dictates the outcome of *L. pneumophila* infection. Conditions that increase iron availability, like iron supplementation of tissue cultures or feeding of iron to animal hosts, favor *L. pneumophila* infection (reviewed in Cianciotto 2007), whereas limiting iron availability attenuates replication (James et al., 1995; Byrd and Horwitz, 2000; Viswanathan et al., 2000).

*L. pneumophila* possesses several systems to salvage and assimilate iron from its environment: the siderophore legiobactin (Liles et al., 2000; Allard et al., 2006), ferric reductase enzymes (Johnson et al., 1991; Poch and Johnson, 1993; James et al., 1997), the FeoB ferrous iron transporter (Robey and Cianciotto, 2002), the *iraAB* locus (Pope et al., 1996; Viswanathan et al., 2000) and two secreted ferric iron reductants, homogenistic acid, and homogenistic acid-melanin (pyomelanin) (Zheng et al., 2013). In addition, both the cytochrome *c* maturation (Ccm) system and cytochrome *c*_4_ are required for expression of the legiobactin siderophore of *L. pneumophila* (Yip et al., 2011). The specific cellular function of Ccm system proteins is to attach heme covalently to cytochrome *c* precursor proteins. The link between Ccm function and iron salvage and assimilation is not unprecedented, since other bacterial species also display this type of metabolic integration (Cianciotto et al., 2005).

The phenotypes of mutants defective in some of the iron salvaging and utilization systems highlight the importance of iron for intracellular replication of *L. pneumophila*. First, mutants defective in iron assimilation (e.g., *feoB, iraA*) exhibit reduced intracellular replication, a defect exacerbated when host cells are pre-treated with iron chelators (Viswanathan et al., 2000; Robey and Cianciotto, 2002). Second, mutants of the *frgA* gene, proposed to encode a homolog of *E. coli* aerobactin siderophore synthetases, are impaired in replication in macrophages. These data indicate a requirement for at least one siderophore during intracellular growth, as legiobactin is dispensable under these conditions (Hickey and Cianciotto, 1997; Allard et al., 2006; Cianciotto, 2007). Third, mutants defective in cytochrome *c*-maturation (*ccm*) exhibit replication defects in macrophages and amoeba (Viswanathan et al., 2002; Yip et al., 2011) and in a mouse pulmonary model of infection (Naylor and Cianciotto, 2004). The defects of *ccm* mutants in macrophages are alleviated by addition of iron and exacerbated by iron depletion (Viswanathan et al., 2002; Naylor and Cianciotto, 2004). Furthermore, studies of *c*-type cytochrome mutants indicate that *L. pneumophila* requires cytochromes *c*_5_ and *c*_1_ to grow in macrophages or amoeba, indicating that poor growth by *ccm* mutants may reflect an inability of *L. pneumophila* to generate the aforementioned cytochromes (Yip et al., 2011). Given the pivotal contributions of *c*-type cytochromes in aerobic respiration, it is easy to surmise why *L. pneumophila* requires these proteins for intracellular growth. Whether *c*-type cytochromes have additional roles during intracellular growth of *L. pneumophila* is an intriguing possibility that is worthy of consideration in light of various additional roles for these proteins discovered in other systems (Yip et al., 2011).

## Host metabolic components exploited by intracellular *L. pneumophila*

### Host amino acid transporters

The notion that *L. pneumophila* absolutely relies on certain host metabolic functions for intracellular replication is demonstrated by an elegant study by Wieland et al. (2005), whereby SLC1A5, a Na^+^/Cl^−^ coupled amino acid transporter of eukaryotes, was identified as necessary for *L. pneumophila* replication in MM6-monocytes. Furthermore, replication by intracellular bacteria required that MM6 cell culture medium be supplemented with amino acids. Analyses of host mRNAs indicated that the SLC1A5 transporter is highly expressed in infected MM6 monocytes. Moreover, pharmacological and siRNA inhibition of the SLC1A5 transporter blocks *L. pneumophila* replication, providing strong evidence that the host amino acid transporter is required for bacterial growth. Thus, by exploiting host amino acid acquisition systems, *L. pneumophila* fuels its own carbon and energy metabolism.

This study also uncovered some differences between the amino acid requirements of intracellular and extracellular bacteria. For instance, L-glutamine supports optimal growth in host cells and in broth cultures (Wieland et al., 2005). Conversely, L-glutamate, L-tyrosine and L-threonine are required for growth in broth cultures but have minimal impact on intracellular growth. From studies of this type, it is possible to predict classes of metabolites that are readily available within host cells.

### The host proteasome

*L. pneumophila* utilizes the Dot/Icm apparatus to translocate a series of effectors across the bacterial plasma membrane. One of these effectors is the AnkB protein (Al-Khodor et al., 2008; Price et al., 2009, 2011; Al-Quadan and Kwaik, 2011; Bruckert et al., 2014). The AnkB protein contains several eukaryotic domains, including an F-box domain (Price et al., 2009; Skaar et al., 2013), which has a well-documented role in ubiquitination. This type of posttranslational modification regulates a variety of cellular processes, such as protein degradation, cell division, and protein trafficking (Schnell and Hicke, 2003; Hershko, 2005; Price et al., 2009; Komander and Rape, 2012).

*L. pneumophila* require AnkB to replicate in various hosts, including U937 macrophages, *A. castellanii*, *A. polyphaga*, as well as for pulmonary infection in a mouse model (Al-Khodor et al., 2008; Lomma et al., 2010; Price et al., 2010). The AnkB effector itself is posttranslationally modified by farnesylation, which mediates its localization on the membrane of the *L. pneumophila* replication vacuole (Price et al., 2010). *L. pneumophila* fails to replicate intracellularly when farnesylation of AnkB is blocked, highlighting the importance of farnesylation and the requirement for the effector in host cells (Price et al., 2009; Al-Quadan and Kwaik, 2011). Anchoring of AnkB to the vacuolar membrane enables the effector to serve as a dock for polyubiquitinated proteins, which are then degraded by the host proteasome (Price et al., 2011). Degradation of polyubiquitinated proteins was deduced to generate a pool of amino acids that support *L. pneumophila* replication within the host (Price et al., 2011). After infection, *L. pneumophila ankB* mutants prematurely differentiate to the transmissive form, as judged by expression of *flaA*, *relA* and *spoT*, three genes that are induced by starvation *in vitro*. However, when the infected cells are supplemented with excess amino acids, *ankB* mutants replicate intracellularly and do not activate the *flaA* promoter. The tight link between host metabolism and the *L. pneumophila* life cycle is further evidenced by the finding that swift farnesylation of AnkB and subsequent assembly and degradation of polyubiquitinated proteins occurs upon attachment of *L. pneumophila* to host cells (Bruckert et al., 2014). This in turn is thought to increase amino acid levels in the host to promote the transition of *L. pneumophila* from the transmissive to the replicative phase of its intracellular life cycle.

### Host phospholipids

The finding that *L. pneumophila* couples fatty acid metabolism to virulence strongly indicated that host lipid or fatty acid metabolites may serve both as nutrients and as signals that relay the metabolic state of the host and the bacterium (Dalebroux et al., 2009; Edwards et al., 2009). Further strengthening this notion is the fact that *L. pneumophila* secretes a number of phospholipase enzymes, some of which have been determined to be virulence factors that act by destroying host membranes (Flieger et al., 2000, 2002, 2004; Bender et al., 2009; Kuhle and Flieger, 2013).

In addition, *L. pneumophila* also synthesizes several effectors that are translocated by the Dot/Icm type IV secretion system that act on phospholipids and modify the phagosomal compartment in which the microbe replicates (VanRheenen et al., 2006; Viner et al., 2012). While much of this work has focused on identification of effectors and their activities, a hypothesis that warrants experimental testing is that *L. pneumophila* deploys effectors not only to alter organelle membranes and signaling pathways in its host, but also to salvage lipid precursors.

### Polyamines

Polyamines, such as spermidine and putrescine, are well known for their effects on cell growth, nucleic acid synthesis, and cell integrity in prokaryotic and eukaryotic organisms (reviewed in Tabor and Tabor 1985; Pegg 1986; Chattopadhyay and Tabor 2013). However, the detailed mechanisms by which these polycationic compounds affect various cellular processes have been the object of ongoing study for several decades and are still being determined.

Microorganisms like *E. coli* and *S. cerevisiae* have served as amenable models for defining the effects of polyamines on cell physiology (Hafner et al., 1979; Geiger and Morris, 1980; Abraham, 1981; Tabor and Tabor, 1985; Chattopadhyay et al., 2009a,b; Chattopadhyay and Tabor, 2013). In *E. coli*, polyamines contribute to optimal aerobic growth, synthesis of proteins and nucleic acids, protection from oxidative stress, and anaerobic growth (Tabor et al., 1980; Chattopadhyay et al., 2009a). A capacity for polyamines to protect *E. coli* from acid stress was recently discovered (Chattopadhyay and Tabor, 2013).

Polyamines have been shown to influence various aspects of microbial pathogenesis (reviewed in Shah and Swiatlo 2008). Of particular interest is that microbes like *Helicobacter pylori* and *Pneumocystis jirovecii* perturb metabolism of polyamines in host cells (Lasbury et al., 2007; Chaturvedi et al., 2012a,b; Xu et al., 2012). The alterations in polyamine metabolism caused by these microbes are thought to trigger apoptosis in the case of *P. jirovecii* and to be linked to *H. pylori*-associated gastric diseases such as cancer.

Polyamines also appear to contribute to replication of intracellular *L. pneumophila*. *L. pneumophila* encodes an HtpB chaperonin (Nasrallah et al., 2011), a protein known in some systems to recruit host mitochondria and alter microfilament arrangements (Chong et al., 2009). In a yeast two–hybrid system, HtpB interacts with eukaryotic S-adenosylmethionine decarboxylase (SAMDC), an enzyme prokaryotes and eukaryotes require for synthesis of spermidine (Caspi et al., 2012). Furthermore, when SAMDC activity is inhibited in U937 macrophages infected with *L. pneumophila*, intracellular replication of the bacterium is significantly reduced (Nasrallah et al., 2011). Given that *L. pneumophila* appears to lack most canonical enzymes required for polyamine synthesis, it has been proposed that *L. pneumophila* salvages any polyamines that promote its replication (Nasrallah et al., 2011). Indeed, exogenous polyamines boost growth of intracellular and extracellular *L. pneumophila*. However, it has not been ruled out that, like other microbial pathogens, *L. pneumophila* may manipulate SAMDC activity to alter the physiology of the host to its advantage. Further studies of the links between host polyamine metabolism and *L. pneumophila* infection promise to increase our understanding of how specific host systems are exploited for microbial pathogenesis.

## Concluding remarks

**Figure 2:**
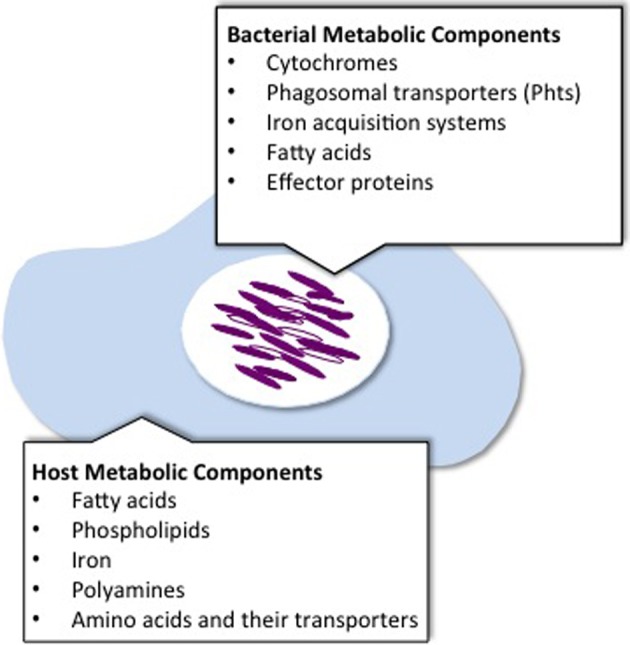
**Metabolic components known to be exploited by *L. pneumophila* for intracellular replication.** To replicate within host cells, *L. pneumophila* exploits metabolic components that may originate from the bacteria or the host.

For a number of years, there has been considerable emphasis on understanding the mechanisms of pathogenesis of intracellular microbes like *L. pneumophila*. Two key aspects of *L. pneumophila* virulence have been object of intense study: (i) the strategies employed by the bacterium to manipulate host cellular processes and (ii) the coupling of its cellular differentiation cycle with virulence. Invariably, both lines of research have discovered intricate connections between microbial metabolism and mechanisms of disease. In several examples presented herein, whether *L. pneumophila* replicates or deploys virulence factors to seek a new host is dictated by the presence of particular metabolites, such as amino acids or fatty acids. In some instances the source of these metabolites is clear (i.e., the host cell), in others, it remains an open question (Figure 2). It is also becoming increasingly clear that at least some of the effectors produced by *L. pneumophila* upon infection are geared toward exploitation of the host as a nutritional niche (Figure 2).

*In vitro* studies have been instrumental in gaining insights to the nutritional requirements and metabolic capabilities of *L. pneumophila*. These together with the experimental approaches taken to dissect the *L. pneumophila* cell differentiation cycle in synchronous broth cultures have laid the foundation to explore further the metabolic strategies employed by *L. pneumophila* to thrive intracellularly. Emerging genetic, molecular and biochemical methodologies increase the number of tools that are available to answer the many questions that remain to be answered regarding the metabolism of *L. pneumophila* in host cells.

### Conflict of interest statement

The authors declare that the research was conducted in the absence of any commercial or financial relationships that could be construed as a potential conflict of interest.
